# Correlates of knowledge on birth defects and associated factors among antenatal mothers in Galle, Sri Lanka: a cross-sectional analytical study

**DOI:** 10.1186/s12884-018-2163-9

**Published:** 2019-01-17

**Authors:** Janithra De Silva, Sujeewa Amarasena, Kapila Jayaratne, Bilesha Perera

**Affiliations:** 1 Department of Community Medicine, Faculty of Medicine, University of Ruhuna, Galle, Sri Lanka; 2 Department of Paediatrics, Faculty of Medicine, University of Ruhuna, Galle, Sri Lanka; 3 Family Health Bureau, Ministry of Health, Colombo, Sri Lanka

**Keywords:** Birth defects, Associated factors, Prevention, Knowledge, Folic acid, Antenatal mothers, Sri Lanka

## Abstract

**Background:**

Birth defects (BD) are considered a leading cause of childhood morbidity and mortality. Personal, cultural, and health care system barriers may increase the incidence of BD in low and middle income countries. In this study we assessed the knowledge of antenatal mothers on BD, associated factors, and prevention and management.

**Methods:**

Three hundred and fifty (350) antenatal mothers were surveyed using a pretested, self-administered questionnaire. The knowledge on BD was evaluated under 3 categories; knowledge on BD, knowledge on associated factors, and knowledge on prevention and management. The total scores were calculated for each category and converted into percentages. A higher percentage score indicates a high level of knowledge. Descriptive statistics and regression models were used for data analysis. Level of significance was considered as *p* < 0.05.

**Results:**

Mean age of the participants was 28.7 years (*SD* = 5.2). The age range was 17–44 years. Most of the participants (79%) had studied up to secondary or tertiary education. The average scores of knowledge on BD, associated factors, and prevention and management of BD were 57.6% (95% CI = 52.3–62.9%), 55.1% (95% CI = 49.8–60.4%) and 58.8% (95% CI = 53.5–64.1%) respectively. The average score on the overall total knowledge was 56.4% (95% CI = 51.1–61.7%). Mother’s level of education, monthly income of the family and number of clinic visits made by the mother were found to be positively associated with the overall knowledge. About 62% of the participants had taken folic acid (FA) preconceptionally, a major preventive factor of BD associated with the nervous system. Folic acid intake was positively associated with age and educational level, but negatively associated with parity. Media (36.9%) and Public Health Midwives (PHMs) (20%) were found to be the major sources of knowledge on BD, associated factors and prevention in this target group.

**Conclusions:**

The average overall knowledge on BD in this group of antenatal mothers was moderate. Thus, there is a need to improve the knowledge in eligible women to reduce the occurrence of BD, ideally before they become pregnant. Media and PHMs were seem to be the effective and possible resources that can be used to educate the community on BD, associated factors and prevention of BD in Sri Lanka.

**Electronic supplementary material:**

The online version of this article (10.1186/s12884-018-2163-9) contains supplementary material, which is available to authorized users.

## Background

Birth defects (BD) are a diverse group of abnormalities of prenatal origin that occur in relation to the structure or function of the individuals [1, 2]. They are well known as a significant and a serious public health problem because of the high morbidity and mortality associated with them [2–4]. Apart from the adverse outcomes in the affected individual, BD impose a huge burden on social, psychological, health aspects and economy of the caregivers and the family. Birth defects are known as a global health problem causing deaths of 303,000 newborns within the first 4 weeks of life each year worldwide [5]. However, the occurrence and the impact of BD are found to be higher in low and middle income countries. More than 94% of serious BD occur in these countries and nearly 95% of the children with severe BD would die eventually [1, 5]. It is said that these differences among developed and developing countries could be partly due to the differences in socio-economic conditions of the individuals and countries, availability of health care facilities and cultural aspects [1]. In addition, lack of knowledge and education of the mothers on BD, the risk factors of BD and the prevention of BD have been identified as major factors which hinder the prevention of BD [1, 2]. Because of the higher morbidity and mortality associated with BD, many countries were not able to achieve the United Nations Millennium Development Goal (MDG) 4 set as to reduce the under-five mortality rate by two-thirds between 1990 and 2015 even though the mortality due to most other causes showed a decline [1, 2].

Sri Lanka, although being a country doing well in maternal and child health, has borne the burden of BD. A study conducted in Sri Lanka in 2014 has found a 4.3% prevalence of birth defects among newborns [6]. Added to that BD have become a leading cause of mortality among children under 5 years in Sri Lanka and the proportional mortality due to BD has increased during the recent years [1, 7–9]. In 2015 BD contributed 46.5% of infant deaths and 36.8% of 1 to 5 year child deaths [8] and in 2016, the contribution for infant and 1–5 year child deaths was 54.5 and 34.5% respectively [9]. Therefore, prevention and proper management of BD has become a priority issue in child health in Sri Lanka. With this background Sri Lanka initiated birth defects surveillance in 2014 and the pilot program was conducted in Galle district.

Though, BD carry a high morbidity and mortality they are considered to be preventable to a large extent [2]. Several interventions such as preconceptional folic acid (FA) supplementation, iodization of food items like salt, immunization with rubella vaccine, screening and treatment of syphilis during pregnancy, identification and management of preexisting health conditions, improvement of nutritional status of the mothers have been each identified as cost-effective preventive strategies of BD [1, 2]. Adding up to these interventions, health education of the mothers and the public has also been identified as a major method in the prevention of BD [1].

Knowledge regarding BD and the management of BD is not only important for the prevention of BD, but for the betterment of the affected individuals as well. The higher the knowledge the parents or the care givers would be having, the higher the chances to reduce the morbidity and mortality associated with BD. Further, the affected children can be saved from adverse social outcomes like social stigmatization and from the extreme consequences like infanticide if mothers are aware on the management options of BD [10]. Higher knowledge on BD, associated factors, prevention and management would invariably minimize the adverse social, psychological, health and economic impacts to the family and eventually the negative impacts to the country.

This study was planned to assess the knowledge of the antenatal mothers on BD, associated factors and prevention and management, and to identify the correlates of such knowledge. We also assessed the preconceptional FA intake and the awareness on the importance of preconceptional FA among the antenatal mothers in Galle, Sri Lanka.

## Methods

### Participants

This study was conducted in Galle district, one of the three districts of Southern Sri Lanka. Health care services are delivered to the community by two teaching hospitals and three base hospitals in the district. Field health care services are delivered by 20 Medical Officer of Health (MOH) units. In 2016, 20,796 pregnancies were estimated and 18,905 live births were reported in Galle district [9].

Antenatal mothers attending in six antenatal clinic centers in a health unit area in Galle district in the Southern province of Sri Lanka were recruited for the study. The mothers who were able to read and understand Sinhala language and who gave consent to participate in the study were recruited using a systematic sampling method.

### Data collection instrument

A self-administered questionnaire was used to collect data. It was pretested using a sample of antenatal mothers attending antenatal clinics in another health unit in Galle district. Please see the attached Additional file 1 for more details.

In the development of the questionnaire for the study, the questionnaire developed by Bello et al., (2013) was used as a reference [3]. Additional questions were added to address the aims of the study. Face validity of the questionnaire was evaluated by two consultant community physicians and two consultant paediatricians.

The questionnaire consisted of two parts; part A to identify the socio-demographic characteristics of the respondents and part B to assess the knowledge of the mothers. In part A of the questionnaire socio-demographic variables including age, highest educational qualification, monthly income, parity, having children with BD, and the number of clinics attended were gathered. Participants were questioned whether they have heard or learnt about BD, and if so from whom or from where they received that information. An added question was included to state whether they had taken preconceptional FA and if so, to state the reason for them to take preconceptional FA supplementation.

In part B of the questionnaire mothers’ knowledge on BD was assessed under 3 sections namely, knowledge on BD, knowledge on the associated factors of BD and knowledge on prevention and management of BD using 10, 21 and 7 statements respectively. In all 3 sections mothers were asked to mark their responses stating whether the given statement is true, false or do not know. A correct response was given a + 1 and an incorrect response or a do not know response was given 0 marks. The total marks were calculated for each category and converted into percentages.

### Procedure

Ethical approval for the study was obtained from the Ethics Review Committee of the Faculty of Medicine, University of Ruhuna, Galle, Sri Lanka. Informed written consent was obtained from the participants after an initial information session. Non – response rate was 2%. The participants who agreed to participate in the study (*n* = 350) were given instructions about the study, the aims and the risks and benefits of the study. They were asked to mark their responses for the statements in the given spaces.

### Data analysis

Data were coded and entered into a database created using the Statistical Package of Social Science (SPSS) version 20.0. Descriptive statistics were used to describe the socio-demographic characteristics and the results were presented as means, frequencies and percentages. The total scores of each category of knowledge were calculated and converted into percentages. Data were analyzed using the SPSS 20 version and *t* test was used to determine the differences between groups. The level of significance was considered as *p* < 0.05. Linear regression models were used to identify the factors associated with the knowledge on BD. To identify the factors associated with the preconceptional FA intake and the knowledge on the importance of preconceptional FA, univariate and multivariate logistic regression analysis were conducted.

## Results

### Socio-demographic characteristics of the participants

A sample of 350 antenatal mothers residing in selected Health Unit was included in the analysis. The majority 313(89.4%) of the participants were Sinhalese while 35(10%) were Muslims and 2(0.6%) were Tamils. The mean age of the participants was 28.7 *(SD* = 5.2) years and the age range was 17 to 44 years. The majority (*n* = 275, 78.6%) have studied above the secondary school; studying beyond the General Certificate of Education of Ordinary Level exam conducted at grade 11 in Sri Lanka and 51(14.6%) out of them have had higher education. Eighty two participants (23.4%) were employed. The monthly income of the majority of the participants (*n* = 166, 47.4%) was between 25,000–49,000 Sri Lankan rupees (140–275 US dollars). There were 14(7.1%) multiparous mothers who already had a child with BD. Of the total, 154(44%) were primiparous mothers. Table 1 shows the socio-demographic characteristics of the participants.

**Table 1:** Socio-demographic characteristics of the participants (*n* = 350)

Demographic characteristic	n	%
Age (years)		
< 25	83	23.7
25–34	220	62.9
≥ 35	47	13.4
Educational level		
Up to secondary school	75	21.4
Above secondary school	224	64.0
Higher education	51	14.6
Monthly income (Sri Lankan Rupees)		
< 25,000	112	32.0
25,000–49,000	166	47.4
≥ 50,000	72	20.6
Parity		
Primiparous	154	44.0
Multiparous	196	56.0
Kids with BD		
Yes	14	4.0
No	336	96.0
Number of antenatal visits		
≤ 5	169	48.3
6–15	163	46.6
≥ 16	18	5.1

### The knowledge on BD and the correlates of knowledge on BD

The average scores of knowledge on BD, associated factors and prevention and management were 57.6% (95% CI = 52.3–62.9%), 55.1% (95% CI = 49.8–60.4%) and 58.8% (95% CI = 53.5–64.1%) respectively. The average score on overall knowledge was 56.4% (95% CI = 51.1–61.7%).

Linear regression analysis results of the predictors of overall knowledge on BD are shown in the Table 2. Mothers’ with higher educational attainment (*p* < 0.001), higher monthly income (*p* = 0.015) and higher number of antenatal clinic visits (p < 0.001) are found to have a higher overall knowledge. Age (*p* = 0.480) and parity (*p* = 0.348) of the mother and having children with BD (*p* = 0.878) were not associated with overall knowledge on BD.

**Table 2:** Linear regression analysis of the effect of socio-demographic characteristics on overall knowledge on BD

Variable	B	SE	t	p
Constant	0.125	0.128	0.973	0.331
Maternal age	0.002	0.002	0.882	0.378
Educational level of the mother	0.118	0.019	6.194	0.000
Monthly income	0.003	0.000	2.455	0.015
Parity	0.018	0.014	1.286	0.199
Having kids with BD	0.010	0.054	0.184	0.854
Total number of clinic visits attended	0.009	0.002	3.953	0.000

### Sources of information on BD among antenatal mothers

In this sample the majority of mothers (*n* = 265, 75.7%) had heard or learnt about BD. Those who had heard about BD and the associated factors had a higher average overall knowledge compared to those who had not (59.6% vs 46.4%, *p* < 0.001). Table 3 shows the sources of knowledge on BD, associated factors, prevention and management.

**Table 3:** The sources of information on BD (*n* = 265)

Source	n	%
Consultant obstetrician	52	6.4
Family doctor	29	3.6
Medical Officer of Health (MOH)	33	4.1
Other doctor	16	1.9
Public Health Midwife (PHM)	162	20.0
Other health care personnel	21	2.6
Printed media	150	18.5
Electronic media	149	18.4
Internet	44	5.4
Parent/relative of a child with BD	14	1.7
School/Higher education institution	63	7.8
Relatives/friends	77	9.5

The major sources of information on BD, associated factors, prevention and management for the antenatal mothers in this target population were Public Health Midwives (PHMs) (20.0%) and the printed (18.5%) and electronic media (18.4%).

### Pre-conceptional folic acid consumption and awareness

Almost two thirds of mothers in the sample (*n* = 218, 62.3%) had taken preconceptional folic acid (FA). Among them the majority (*n* = 146, 70.0%) were in the age group 25 to 34 years. The majority (*n* = 185, 84.9%) had studied above secondary school level and 44 (23.8%) among them had higher education. Out of the total who had FA preconceptionally, 110 (50.4%) were primiparous mothers, which is a 71.4% of the total number of primiparous mothers in the sample. Among the multiparous mothers (*n* = 196) in the study sample only 55.1% have taken preconceptional FA.

Results of the univariate and multivariate regression analysis used to identify the factors associated with the practice of preconceptional FA intake are shown in Table 4.

**Table 4:** Univariate and multivariate analysis of the effect of socio-demographic factors on preconceptional FA intake (*n* = 350)

Characteristic	Univariate analysis			Multivariate analysis		
	OR^b^	95% CI	*p* value	OR^b^	95% CI	*p* value
Age (years)						
≤ 24	2.0	1.2–3.4	0.007	2.1	1.2–3.7	0.009
25–34^a^						
≥ 35	1.0	0.5–2.0	0.957	0.8	0.4–1.5	0.437
Monthly income (Sri Lankan Rupees)						
≤ 24,000	3.4	1.7–6.7	0.000	2.0	0.9–4.2	0.072
25–49,000	2.4	1.2–4.6	0.009	1.9	1.0–3.8	0.049
≥ 50000^a^						
Educational level of the mother						
Up to secondary school	2.6	1.6–4.4	0.000	2.0	1.1–3.6	0.019
Above secondary school^a^						
Parity						
Primiparous^a^						
Multiparous	2.0	1.3–3.2	0.002	2.4	1.4–3.9	0.001

^a^Reference category , ^b^OR- Odds Ratio

According to the univariate analysis mothers aged 25 years or more (*p* < 0.01), mothers with higher educational attainment (*p* < 0.01) and higher socio-economic status (*p* < 0.01) and primiparous mothers (*p* < 0.01) were more likely to have preconceptional FA supplementations. Multivariate regression analysis showed, the higher maternal age (*p* < 0.01) and higher educational level of the mother (*p* < 0.05) and lower parity (*p* < 0.01) as the factors associated with the preconceptional FA intake among participants in the study sample.

The mothers who had taken FA during their pre-pregnant period stated the reason for them to take FA. The reasons stated by the mothers are shown in Table 5.

**Table 5:** The reasons for mothers to take pre-conceptional folic acid (*n* = 218)

Reasons	n	%
To prevent the defects associated with the nervous system of the fetus/ baby	11	5.0
For the development of the nervous system of the fetus/ baby	78	35.8
Taken but do not know the reason	31	14.2
Non-specific/ incorrect or invalid reason	98	45.0

Among the mothers who had taken preconceptional FA, only 11(5.0%) mothers correctly stated the importance of preconceptional FA as prevention of BD associated with the nervous system of the fetus. Another 78(35.8%) mothers stated that FA is important for the development of the nervous system of the fetus. The rest 129(59.2%) were not aware about the importance of preconceptional FA in reducing BD.

Results of the univariate analysis of the association of socio-demographic factors on the awareness on the importance of preconceptional FA intake is shown in the Table 6. The mothers who stated that FA prevents BD and the mothers who stated that FA is important for the development of nervous system were taken collectively as the group aware on the importance of preconceptional FA.

**Table 6:** Univariate analysis of the effect of socio-demographic factors on the correct knowledge on the importance of preconceptional FA (*n* = 218)

Characteristic	OR^b^ (%)	95% CI	*P* value
Age (Years)			
25–34^a^			
≤ 24	0.9	0.4–1.7	0.688
≥ 35	1.1	0.5–2.4	0.861
Monthly income (Rupees)			
≤ 24,000	0.9	0.4–1.9	0.825
25–49,000	1.0	0.6–1.9	0.985
≥ 50000*			
Educational level of the mother			
Up to secondary school	0.9	0.4–2.0	0.839
Above secondary school^a^			
Parity			
Primiparous	1.4	0.9–2.5	0.177
Multiparous^a^			
Number of clinic visits			
Up to 5	0.7	0.2–2.5	0.628
6–15	0.3	0.1–1.0	0.046
≥ 16^a^			
Heard about BD			
Yes	0.4	0.2–0.9	0.018
No^a^			

^a^Reference category, ^b^OR - Odds Ratio

The awareness on the importance of preconceptional FA intake was not associated with any of the socio-demographics we analyzed. However, the mothers who have learnt or heard about BD demonstrated a better knowledge on the importance on preconceptional FA compared to those who had not heard about BD.

## Discussion

The knowledge on BD among the antenatal mothers in Galle, Sri Lanka was found to be moderate in all the components namely; knowledge on BD, knowledge on associated factors and knowledge on prevention and management. The overall knowledge on BD was 56.4%. This stresses the urgent need for Sri Lanka to pay more attention towards public awareness on BD, associated factors, prevention and management in order to reduce BD in the country in an era where BD cause a significant morbidity and mortality.

The mothers with higher educational attainment, mothers who were from wealthier families and mothers who had sought more antenatal care or had a higher number of clinic visits reported a better knowledge in all the components compared to the others. However, age, parity and having a child with BD did not show any association with the overall knowledge on BD. A study conducted in Ghana found that age, educational level, parity and number of clinic visits did not have a significant relationship with the knowledge on BD [3]. Studies done in Nigeria and Iran found a positive relationship between the knowledge on BD and the level of education which is consistent with the results of the present study [10–12]. A study done in Nigeria found a positive association between BD knowledge and  age, social class, religion practiced and the location of the antenatal clinic center [12]. They stated that mothers who had received care from a tertiary hospital have had a better knowledge on BD compared to mothers who had received care from a local hospital [12]. The present study also was carried out at a community health area close to a medical faculty and two tertiary care hospitals. Therefore, the knowledge regarding BD could be higher among mothers who participated in the present study.

Our results indicate that mothers who had made more antenatal visits to health care workers or clinics have a higher overall knowledge on BD. Therefore, the primary health care workers of Sri Lanka should ensure the attendance of the antenatal mothers at least for the minimum number of clinics scheduled for the mothers. Special attention should be paid for the mothers with low educational and low socio-economic status. However, the number of antenatal visits depends on the period of gestation of the mothers. In the current practice of Sri Lanka, the antenatal mothers should make a minimum of nine field antenatal clinic visits and should receive three domiciliary visits in an uncomplicated pregnancy [13]. The number of field visits and consultations by the consultant obstetrician depends on the risk assessment of the mother [13]. It also varies according to the health seeking behavior of the mother. In contrast to the results of the present study, several studies have shown that there is no association between the number of antenatal visits and the knowledge on BD [3, 11]. This difference in knowledge could be due to the routine nature of the antenatal clinics, the format of the antenatal sessions and the topics discussed at the antenatal clinics in the study setting. This might have been influenced by the health seeking behavior of mothers in the study area. Therefore, the antenatal care services and the health seeking behavior of the mothers in Galle, Sri Lanka seem to be effective in increasing knowledge on BD. Since the mothers should possess knowledge on BD ideally before they become pregnant, health education about BD should be commenced during the preconceptional period rather than during the antenatal period.

In this study group 62.3% (*n* = 218) mothers have taken pre-conceptional FA. It is satisfactory compared to a study done in Kandy, Sri Lanka where preconceptional FA consumption among two groups; mothers with and without children with neural tube defects (NTD) was 0% and 13% respectively [14]. Many researchers have found a low preconceptional FA intake among mothers in many communities [15–17]. Some studies stressed on the low preconceptional FA intake even though many participants have heard about FA [15, 17–19]. A study done in Taiwan, found that preconceptional FA intake was only 15.6%, though nearly 90% of the sample of women was aware of folic acid [18]. It states about a possibility of these mothers getting aware of FA after they become pregnant, since the study was conducted among the antenatal mothers. A study done in Pakistan found that very few had received FA supplementation during pre-pregnancy and pregnancy period. According to them, only 51.25% had received FA even during pregnancy [20].

We found that the preconceptional FA intake is positively associated with higher maternal age, higher educational level and lower parity of the mother. Nilsen and collegues also found a better preconceptional FA intake associated with higher maternal age, higher education, and lower parity which is consistent with our study [16]. Several other studies also found that preconceptional FA intake is higher among mothers with higher education [17, 19–21]. Therefore, Sri Lanka should pay further attention on education of the adolescent girls in the country. Within the education systems knowledge on reproductive health should be disseminated including the knowledge on BD and prevention. Through that, positive health behaviors like preconceptional FA intake by eligible females can be expected while increasing the awareness on BD, associated factors and prevention. Then Sri Lanka can achieve a reduction of BD, along with the associated morbidity and mortality.

This study revealed that preconceptional FA intake is higher among primiparous mothers (*p* < 0.01). In Sri Lanka, a preconception care package was introduced in 2012 as a part of the maternal care package for the Sri Lankan mothers, particularly targeted for newly married couples [9] and may be an explanation for the higher proportion of primiparous mothers taking preconceptional FA identified in our study results. Therefore, this finding of our study stresses the necessity of care before the subsequent pregnancies.

Among the mothers who have taken preconceptional FA only 5.04% stated that it prevents the BD of the nervous system of the baby and another 35.8% stated that it is needed for the development of the nervous system of the baby. About 14.2% (*n* = 31) were not aware of the importance of preconceptional FA and the rest 44.9% (*n* = 98) stated nonspecific or incorrect reasons on the importance of preconceptional FA. This finding stresses the fact that even the women who took FA preconceptionally, have inadequate knowledge about the importance of FA. If the mothers were aware on the NTD prevention associated with FA, the preconception FA intake would more likely to be much higher. Therefore, when prescribing FA to the eligible females it is important to highlight the importance of preconceptional intake of FA. Dissanayake and collegues concluded on a grossly inadequate knowledge on FA in Sri Lanka, even among the mothers having children with NTD [14]. Many studies have revealed inadequate awareness about the importance of FA and stressed on the importance of public health strategies to increase awareness on FA [14, 15, 19–23]. However, countries like Israel and Canada have shown a higher prevalence of folate awareness [19]. It would be better to look for the methods they have used to achieve these targets when planning public health strategies to increase awareness on FA among Sri Lankan mothers.

According to the results of the present study no association between  the socio-demographic factors and the knowledge on the importance of preconceptional FA was elicited. Our finding is consistent with the results derived by Nosrat and collegues [15]. However, many studies showed associations between the awareness on FA and socio-demographics. An Ethiopian study revealed that women with better family income were more aware of the importance of FA [21]. Many other studies revealed a better awareness on FA among females with higher educational attainment [17, 19, 21, 22]. This might be an evidence for the fact that there is a lack in the education curriculums of Sri Lanka with regard to certain health aspects like BD prevention.

Prevention of NTD is highly associated with the “preconceptional consumption” of FA since the neural tube develops by the 28th day of gestation, a time period where the mother herself is not aware about the pregnancy [17, 18, 24]. Two major methods, namely; creating awareness on FA and supplementation of FA among the females in the child bearing age have been identified as main preventive strategies against NTD [18]. In the supplementation of folic acid for females in childbearing age, fortification plays a major role and the other method of supplementation is tablets containing FA [2, 25]. Jou and collegues cited that fortification has shown a 31 and 16% reduction in the prevalence of spina bifida and anencephaly respectively in the United States once US has fortified the food with FA [18]. Many other countries like Canada, Costa Rica, Chile and South Africa also have shown a significant reduction of NTD after food fortification with FA [2]. In countries like Sri Lanka, where such a policy is not being practiced yet [2], making the public aware on the importance of FA through health education would be the best method to reduce the NTD, which showed a prevalence of 1.4 per 1000 live births [14]. With a better awareness on the importance of FA, preconception FA intake would be increased in the country. When making plans to make the eligible females aware of the importance of FA, it is important to make them aware from their adolescence while they are in the schools to have a better coverage.

Many researchers have investigated for the methods of acquiring knowledge about BD and FA among the communities. Many revealed mass media and health care workers as the main sources of information on BD and FA [12, 17, 22]. When considering the methods of acquiring knowledge about BD in the present study, PHM, printed and electronic media were the leading sources of knowledge to the mothers. Dissanayake and collegues  also stated family health worker as the main source of information on FA to the mothers followed by media [14]. This may be due to the PHMs’ role as the grass root level health care workers in the delivery of field maternal and child care services in Sri Lanka. It might be further strengthened by the close relationship between the Sri Lankan mothers and the PHMs. This finding gives a positive feedback to initiate the BD prevention programmes through the PHMs, a well-established, strong platform with proven results in uplifting the maternal and child health in the country. Wide availability of electronic media like television and radio also has played a major role in delivering health messages to the community of Sri Lanka.

However, the knowledge on BD, gained through a medical officer is not satisfactory  in this target group although in some studies medical practitioners were found to be the major source of information [18, 19]. Therefore, public awareness through medical practitioners should be given more attention in Sri Lanka in order to prevent BD. A study conducted in Poland found internet as the major source of information for the participants of the study [26]. This would be useful for Sri Lanka as well because the knowledge can be disseminated through internet and social media. This can be applied especially to the teenagers; the next generation of parents, among whom social media and internet usage is highly prevalent [27]. Some studies have tested and proved the effectiveness of various health education methods and materials with regard to prevention of certain causes of BD [28–31]. Many researchers have targeted on the awareness and intake of FA [28–30]. The “Promotora de Salud model” which relied on interpersonal connections of the community health workers, has shown an increased awareness and practice of FA among the participants in several studies [28, 29]. Sri Lanka also can practice this method through PHMs. Effectiveness of health education through village clinics, written materials and text messages was shown in a study conducted among rural Chinese women [30]. A study done in Poland to assess the impact of health education on knowledge and prevention behaviour of congenital toxoplasmosis has stressed the necessity of modern promotional technologies apart from the traditional written education materials [31].

For Sri Lanka, prevention of BD is a key priority in child health due to the fact that BD associated mortality is a leading cause for infant and 1–5 year child mortality. If the public is much aware about the gravity of BD, they would be interested in learning about BD and their prevention. According to a Nigerian study, 86.5% of the respondents believed that more public education can reduce the occurrence of BD [4]. Therefore, public awareness through health education seems to be one of the major methods and a key priority in the prevention of BD in Sri Lanka. When planning the health education programs on BD in Sri Lanka, methods like education through PHMs and medical officers, distribution of pamphlets among the eligible females, giving messages through the electronic and social media and text (SMS) and video (MMS) messages need to be considered and implemented without any delays.

We gathered data from a sample of 350 antenatal mothers which is relatively a large sample. Data was collected from six field antenatal clinic centers. Data collection was performed by a single investigator which would have increased the consistency in data collection approach. These were the strengths of the study.

There were few limitations to the study. This study was confined to one health unit area. We were not able to recruit few (less than 5 %) participants from ethnic groups other than Sinhalese due to their inability to read and write Sinhala language. Also due to the self-administered nature of the questionnaire mothers might have understood the responses with minor variations.

## Conclusions

The knowledge about BD, associated factors, prevention and management among antenatal mothers was found be moderate in this study population. The knowledge on the importance of preconceptional FA is found to be inadequate. Therefore, effective health education programs like education through PHMs and medical officers, distribution of pamphlets among the eligible females, giving messages through electronic and social media and mobile phones need to be developed and implemented. Printed and electronic media and the PHMs seem to be the effective sources that can be used to disseminate knowledge on BD. Medical practitioners should pay more attention towards health education of their clients on BD and prevention of BD. They should target especially on cost effective interventions like preconception FA supplementation, nutrition education and infection prevention. It is needed to emphasize the importance of FA when prescribing FA to the eligible females in order to have a better compliance. When delivering preconception care services, care before the subsequent pregnancies also should be considered.

## Additional file

Additional file 1: Questionnaire to assess the knowledge of the antenatal mothers on birth defects, associated factors, prevention and management. This is a self-administered questionnaire used to collect data from the antenatal mothers. In the development of the questionnaire for the present study, the questionnaire developed by Bello et al., (2013) was used as a reference [3]. Additional questions were added to address the aims of the study. The questionnaire consisted of two parts; part A and part B. Part A of the questionnaire was used to gather socio-demographic data including age, ethnicity, highest educational qualification, monthly income, parity, previous children with birth defects, and the number of clinics attended. Data was obtained on prior awareness on birth defects and the sources of such information. An added question was included to inquire on the preconceptional FA intake. If FA was taken preconceptionally, the reason for them to take preconceptional FA supplementation was inquired. Part B of the questionnaire consisted of three sections; knowledge on BD, knowledge on associated factors of BD and knowledge on prevention and management of BD. Respectively, 10, 21 and 7 statements were included in each section. In all 3 sections mothers were asked to mark their responses stating whether the given statement is true, false or do not know. (DOCX 22 kb)

## Abbreviations

BD: Birth defects
FA: Folic acid
NTD: Neural tube defects
PHM: Public Health Midwife
