# Supplementary material for: Correlates of knowledge on birth defects and associated factors among antenatal mothers in Galle, Sri Lanka: a cross-sectional analytical study
Source: BMC Pregnancy Childbirth. 2019 Jan 17;19:35. doi: 10.1186/s12884-018-2163-9 (PMC6337825; doi:10.1186/s12884-018-2163-9)
Supplement: Supplementary file 1 — Questionnaire to assess the knowledge of the antenatal mothers on birth defects, associated factors, prevention and management. This is a self-administered questionnaire used to collect data from the antenatal mothers. In the development of the questionnaire for the present study, the questionnaire developed by Bello et al., (2013) was used as a reference [3]. Additional questions were added to address the aims of the study. The questionnaire consisted of two parts; part A and part B. Part A of the questionnaire was used to gather socio-demographic data including age, ethnicity, highest educational qualification, monthly income, parity, previous children with birth defects, and the number of clinics attended. Data was obtained on prior awareness on birth defects and the sources of such information. An added question was included to inquire on the preconceptional FA intake. If FA was taken preconceptionally, the reason for them to take preconceptional FA supplementation was inquired. Part B of the questionnaire consisted of three sections; knowledge on BD, knowledge on associated factors of BD and knowledge on prevention and management of BD. Respectively, 10, 21 and 7 statements were included in each section. In all 3 sections mothers were asked to mark their responses stating whether the given statement is true, false or do not know. (DOCX 22 kb) [file 12884_2018_2163_MOESM1_ESM.docx]

**Questionnaire**

**Knowledge of the Antenatal Mothers on Birth Defects, Associated Factors and Prevention and Management**

*Dear participant,*

*This is a questionnaire set to assess the knowledge of the antenatal mothers on birth defects (BD), associated factors of BD and prevention and management of BD. Please answer the questions to the best of your knowledge. We appreciate your participation in this study.*

**Part A**

**Please provide answers within the given space or put a tick for the correct response.**

1. Age (in years): …………

2. Highest educational level:

| No schooling |  |
| --- | --- |
| Up to grade 5 |  |
| Up to grade 11 |  |
| GCE/OL passed |  |
| Up to GCE advanced level |  |
| GCE/AL passed |  |
| Higher education |  |

3. Ethnicity:

i. Sinhalese □ ii. Tamil □

iii. Christian □ iv. Muslim □

4. Occupation: …………………………..

5. Occupation of the spouse: …………………………………

6. Monthly income: Rs: ……………………………………………

7. Parity

i. 1 □ ii. 2 □ iii. 3 □

iv. 4 □ v. 5 □ vi. >5 □

8. Do you have children with birth defects? Yes □ No □

9. State the number of clinic visits attended during the current pregnancy.

i. Clinics conducted by consultant obstetrician -

ii. Hospital clinics - ……………..

iii. Field antenatal clinics - …………………..

iv. Other (please specify) - …………………………

10. Have you heard about birth defects previously? Yes □ No □

11. If the answer for question 8 is “YES”, state the source of information. (Put a tick “√” in front of the relevant category) – You can have more than one response.

| **Source** |  |
| --- | --- |
| Consultant obstetrician |  |
| Family doctor |  |
| Medical Officer of Health (MOH) |  |
| Other doctor |  |
| Public Health Midwife (PHM) |  |
| Other health care personnel (eg.nurses) |  |
| Printed media |  |
| Electronic media |  |
| Internet |  |
| Parent / relative of a child with BD |  |
| School/ Higher education institution |  |
| Relatives/ friends |  |
| Other (please specify) |  |

12. Did you take folic acid preconceptionally? Yes □ No □

13. If your answer for question 12 is “yes”, please state the reason for you to take folic acid preconceptionally.

………………………………………………………………………………………………………………………………………………………………………………………………………………………………………………………………………………………………………………………………………………………………………………………………………………………………

**Part B**

Section I

Please mark your response to state whether the given statements on birth defects are true, false or do not know. Put a √ in the relevant cage.

| Birth defects | True | False | Do not know |
| --- | --- | --- | --- |
| are diseases acquired by pregnant females. |  |  |  |
| are defects occurring during the fetal life. |  |  |  |
| can be seen as external abnormalities. |  |  |  |
| can present as defects or malformations of internal  organs. |  |  |  |
| can be seen as psychological disorders. |  |  |  |
| can be seen as alterations in activities/ behaviours. |  |  |  |
| can occur if a pregnant mother is contacted with another  child affected with BD. |  |  |  |
| occur because of the adverse planetary influences of the  mother. |  |  |  |
| can occur among the other children of the mother if she  has a child with birth defects. |  |  |  |
| can be detected later as well as soon after the birth. |  |  |  |

Section II

Please mark your response to state the associated factors for birth defects. Put a √ in the relevant cage

| The factors associated with birth defects are, | True | False | Do not know |
| --- | --- | --- | --- |
| consanguinity of the parents |  |  |  |
| mother being subjected to malevolent charms |  |  |  |
| genetic mutations of the fetus |  |  |  |
| maternal age more than 40 years |  |  |  |
| nutritional deficiencies of the mother |  |  |  |
| obesity of the mother |  |  |  |
| adverse planetary influences of the mother |  |  |  |
| chronic illnesses of the mother |  |  |  |
| long-term medication taken by the mother |  |  |  |
| infections of the mother during pregnancy |  |  |  |
| intake of medication by the mother during pregnancy  without a prescription of a medical officer |  |  |  |
| psychological problems of the mother |  |  |  |
| maternal smoking before pregnancy |  |  |  |
| maternal smoking during pregnancy |  |  |  |
| maternal alcohol consumption during pregnancy |  |  |  |
| maternal exposure to X-rays or any other radiation  during pregnancy |  |  |  |
| maternal intake of food items that are forbidden by  the elders |  |  |  |
| supernatural forces of the universe |  |  |  |
| becoming pregnant 3 months within immunization  with rubella vaccine |  |  |  |
| maternal exposure to chemicals or toxic gases |  |  |  |
| mother getting contacted with dogs’ or cats’ feces  during pregnancy |  |  |  |

Section III

Please mark your response to state whether the given statements on prevention and management of birth defects are true, false or do not know. Put a √ in the relevant cage.

| Statement | True | False | Do not know |
| --- | --- | --- | --- |
| Many birth defects are preventable. |  |  |  |
| Can be cured by healing rituals. |  |  |  |
| Intake of iodized salt reduces the risk of BD. |  |  |  |
| Preconceptional folic acid intake prevents the  occurrence of BD. |  |  |  |
| Many of the defects are treatable with medical and  surgical management. |  |  |  |
| Physiotherapy is used in the management of birth  defects or the complications of BD. |  |  |  |
| Will be corrected automatically once the adverse  planetary influences wear off. |  |  |  |

*[The questionnaire developed by Bello et al, (2013) was used as a reference questionnaire]*

[Bello AI, Acquah AA, Quartey NA, Hughton A. Knowledge of pregnant women about birth defects. BMC Pregnancy Childbirth. 2013; 13:45.]
